# Highly Efficient and Stable FAPbI_3_ Perovskite Solar Cells and Modules Based on Exposure of the (011) Facet

**DOI:** 10.1007/s40820-023-01103-8

**Published:** 2023-05-28

**Authors:** Kai Zhang, Bin Ding, Chenyue Wang, Pengju Shi, Xianfu Zhang, Cheng Liu, Yi Yang, Xingyu Gao, Rui Wang, Li Tao, Keith G. Brooks, Songyuan Dai, Paul J. Dyson, Mohammad Khaja Nazeeruddin, Yong Ding

**Affiliations:** 1 Beijing Key Laboratory of Novel Thin-Film Solar Cells, North China Electric Power University, Beijing, 102206 People’s Republic of China; 2 Institut des Sciences et Ingénierie Chimiques, Ecole Polytechnique Fédérale de Lausanne (EPFL), Lausanne, 1015 Switzerland; 3 Shanghai Synchrotron Radiation Facility (SSRF), Shanghai Advanced Research Institute, Chinese Academy of Sciences, Shanghai, 201204 People’s Republic of China; 4 School of Engineering, Westlake University and Institute of Advanced Technology, Westlake Institute for Advanced Study, Hangzhou, 310024 People’s Republic of China; 5 Hubei Yangtze Memory Laboratories, Wuhan, 430205 People’s Republic of China; 6 School of Microelectronics, Hubei University, Wuhan, 430062 People’s Republic of China

**Keywords:** Renewable energy, Perovskite solar cell, Perovskite solar module, Facet engineering

## Abstract

Perovskite crystal facets greatly impact the performance and stability of their corresponding photovoltaic devices. Compared to the (001) facet, the (011) facet yields better photoelectric properties, including higher conductivity and enhanced charge carrier mobility. Thus, achieving (011) facet-exposed films is a promising way to improve device performance. However, the growth of (011) facets is energetically unfavorable in FAPbI_3_ perovskites due to the influence of methylammonium chloride additive. Here, 1-butyl-4-methylpyridinium chloride ([4MBP]Cl) was used to expose (011) facets. The [4MBP]^+^ cation selectively decreases the surface energy of the (011) facet enabling the growth of the (011) plane. The [4MBP]^+^ cation causes the perovskite nuclei to rotate by 45° such that (011) crystal facets stack along the out-of-plane direction. The (011) facet has excellent charge transport properties and can achieve better-matched energy level alignment. In addition, [4MBP]Cl increases the activation energy barrier for ion migration, suppressing decomposition of the perovskite. As a result, a small-size device (0.06 cm^2^) and a module (29.0 cm^2^) based on exposure of the (011) facet achieved power conversion efficiencies of 25.24% and 21.12%, respectively.

**Supplementary Information:**

The online version contains supplementary material available at 10.1007/s40820-023-01103-8.

## Highlights

- The (011) facet has excellent charge transport properties achieving closer alignment of energy levels.
- By exploiting the (011) facet a device (0.06 cm^2^) and a module (29 cm^2^) achieved power conversion efficiencies of 25.24% and 21.12%, respectively.

**Supplementary Information:**

The online version contains supplementary material available at 10.1007/s40820-023-01103-8.

## Graphical abstract

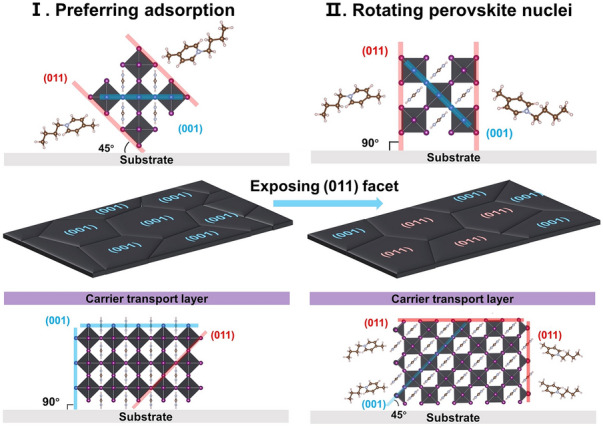

## Introduction

Perovskite solar cells (PSCs) have been studied extensively in the past decade, with a certified record power conversion efficiency (PCE) of 25.7% recently reported [1–3]. However, the PCE of perovskite solar modules (PSMs) decreases rapidly with increasing module size, and the efficiency of mini-modules (less than 200 cm^−2^) is generally < 20% [4, 5]. Facets with different atomic arrangements and coordination impact the physicochemical properties of perovskites, such as carrier transport, surface energy level and stability [6–13]. Recently, the (111) facet in FAPbI_3_ films has been demonstrated to improve the efficiency and stability of PSCs due to high carrier mobility, low exciton binding energies and thermodynamic stability [8, 9]. The perovskite single crystal provides a platform to investigate the photoelectric properties of different facets [6]. Devices fabricated on the (110) facet of MAPbBr_3_ single crystals showed a 153% improvement of responsivity compared to those fabricated on the (100) and (110) facets of MAPbI_3_ single crystals. Devices based on the (110) facet of MAPbBr_3_ exhibited a lower dark current and enhanced photoresponse than other facets, which can be attributed to anisotropic arrangements and polarization differences [14, 15]. Therefore, it has been proposed that the (011) facet of FAPbI_3_ should have excellent photoelectric performance, with the potential for enhancing the photovoltaic performance and stability of PSCs. However, a significant obstacle to exposing (011) facets in FAPbI_3_ films is that the growth of the (011) plane is energetically unfavorable due to the influence of methylammonium chloride (MACl) additive [16–18]. Compared to the (011) plane of FAPbI_3_, the (001) plane has a higher atom density, and MACl is more likely to incorporate into the (001) plane, reducing the surface energy of (001) planes more significantly than that of (011) planes. Controlled by thermodynamics, FAPbI_3_ crystals grow along the (001) plane and the (011) facet disappears.

Dopant engineering is the most common strategy used to modulate facets of perovskite crystals [7–9, 19–21]. By using 3-(decyldimethylammonio)-propane-sulfonate as a dopant to change the growth rate of different facets of MAPbI_3_, single crystals were tuned from (100) and (112) to (002) and (110), respectively [20]. Additionally, butylamine hydroiodide dopant triggered the transition of the MA_3_Bi_2_I_9_ perovskite facet from (001) to (110) by decreasing the surface energy of the (011) facet [21]. Surface-anchoring alkylamine dopant was used to restrict the tilt of grains during growth, resulting in the formation of a (001) facet-dominant polycrystalline perovskite film. Based on this film, the device achieved a PCE of over 23% and demonstrated good operational stability [19]. Notably, a FAPbI_3_ polycrystalline film assembled from single crystals with well-defined (001) and (111) facets was obtained using piperidine dopant [8, 9].

Here, we fabricated FAPbI_3_ perovskite films doped with 1-butyl-4-methylpyridinium chloride ([4MBP]Cl), that results in the exposure of (011) facets, leading to improved charge carrier transport and energy level alignment. The [4MBP]Cl-doped perovskite films have fewer defects, suppressing non-radiative recombination. Photovoltaic devices and modules employing the perovskite with exposed the (011) facets exhibit outstanding PCEs and operational stability.

## Experimental Sections

### Materials

Formamidinium iodide (FAI) and phenethylammonium iodide (PEAI) were purchased from GreatCell. PbI_2_ and 1-butyl-4-methylpyridinium chloride ([4MBP]Cl) were purchased from TCI. Spiro-OMeTAD, MACl and CsCl were obtained from Xi’an Polymer Light Technology. Isopropyl alcohol and acetone were purchased from Sinopharm Chemical Reagent Co., Ltd. Dimethylformamide (DMF), dimethyl sulfoxide (DMSO), chlorobenzene (CB), 4-tert-butylpyridine (tBP), bis(trifluoromethane)sulfonimide lithium salt (Li-TFSI) and FK209 Co(III) TFSI salt (Co-TFSI) were purchased from Sigma-Aldrich. Titanium tetrachloride (TiCl_4_) and stannous chloride dehydrate (SnCl_2_·2H_2_O) were purchased from Sinopharm Chemical Reagent Co., Ltd (China).

### Small-size Device Fabrication

Small-size devices were fabricated using a planar architecture comprising FTO/compact TiO_2_ layer (c-TiO_2_)/compact SnO_2_ layer (c-SnO_2_)/perovskite/Spiro-OMeTAD/Au. The patterned FTO substrate (Asahi FTO glass, 12–13 Ω cm^−2^) was sequentially cleaned with detergent (5% Hellmanex in water), deionized water, acetone and isopropanol in an ultrasonic bath for 30 min. The c-TiO_2_ and c-SnO_2_ layers were prepared by chemical bath deposition (CBD) [22]. Then, the prepared FTO/c-TiO_2_/c-SnO_2_ substrates were transferred to a glove box for the deposition of the perovskite. The 1.4 M FAPbI_3_ perovskite precursor (PbI_2_:FAI = 1.05:0.95) was dissolved in DMF:DMSO = 7:3 (volume ratio) and 0.8 mol% MAPbBr_3_, 5 mol% CsCl and 30 mol% MACl were added to the perovskite precursor solution. [4MBP]Cl was added at different molar ratios (0, 0.3, 0.5 and 0.7 mol%) to the perovskite solution. The perovskite precursor was spin-coated on the c-TiO_2_/c-SnO_2_ substrates with a two-step process. The first step was 1000 rpm for 10 s with an acceleration of 200 rpm s^−1^. The second step was 5,000 rpm for 30 s with an acceleration of 1,000 rpm s^−1^. 20 s into the second spinning step, 100 µL of CB anti-solvent was drop casted. The perovskite film was sequentially heated to 100 °C for 30 min and 150 °C for 10 min. For the post-treatment, 40 µL of PEAI in isopropanol (5 mg mL^−1^) was deposited on the perovskite films at 5,000 rpm for 30 s, and 30 µL of Spiro-OMeTAD (90 mg mL^−1^ dissolved in CB) doped with tBP, Li-TFSI (520 mg mL^−1^ in acetonitrile), and Co-TFSI (300 mg mL^−1^ in acetonitrile) was deposited on top of the perovskite layer by spin-coating at 4000 rpm for 30 s. A ~ 70-nm gold electrode was then deposited on the films by thermal evaporation.

Fabrication of c-TiO_2_ layer: The cleaned FTO substrate was placed in a sealed glass container and immersed in an aqueous TiCl_4_ solution, prepared by mixing a 2-M aqueous TiCl_4_ solution with deionized water in a 1:10 molar ratio. The 2-M aqueous TiCl_4_ solution was prepared by dissolving TiCl_4_ in deionized water at 0 °C. The glass container containing the FTO substrate was placed in a drying cabinet at 70 °C for 1 h. After cooling, the FTO/c-TiO_2_ substrate was thoroughly rinsed with ethanol and deionized water, and then dried at 120 °C for 1 h.

Fabrication of c-SnO_2_ layer: The FTO/c-TiO_2_ substrate was placed in a sealed glass container and immersed in a solution consisting of 2 M-SnCl_2_ in ethanol and deionized water, with a molar ratio of 1:50. The 2-M-SnCl_2_ ethanol solution was prepared by dissolving SnCl_2_·2H_2_O in ethanol. Then, the glass container was placed in a drying cabinet at the temperature of 70 °C for 1 h. After cooling, the FTO/c-TiO_2_/c-SnO_2_ substrate was thoroughly rinsed with ethanol and deionized water, and subsequently annealed on a hotplate at 190 °C for 1 h.

### Module Fabrication

PSMs with 8 sub-cells connected in series were fabricated on FTO glass substrates with a size of 6.5 cm × 7.0 cm. The series interconnection of the module was realized by P1, P2 and P3 lines, which were patterned using a laser scribing system with 1064 nm of Nd:YAG/Nd:YVO4 lasers and a power of 20 W (Trotec). The FTO substrate was pre-patterned for P1 (with a width of 40 μm) by means of 60% laser power at a speed of 300 mm s^−1^ with a frequency of 65 kHz and pulse width of 120 ns. The subsequent processes for the preparation of c-TiO_2_/c-SnO_2_ substrates were the same as for the small-area devices as described above. The perovskite precursor deposition and fabrication procedures were also similar to those of the small-size solar cells except for the concentration of perovskite precursor. 1.3 M of perovskite precursor was employed to make the perovskite layer by spin-coating and the custom-made gas-induced pump method. The perovskite precursor was spin-coated on the c-TiO_2_/c-SnO_2_ substrates using a two-step process. The perovskite films were annealed at 100 ºC for 1 h and 150 ºC for 10 min. After cooling to room temperature, the procedures to spin-coat the PEAI passivation layer, and the spiro-OMeTAD layer were similar with those of the small-size devices. The P2 lines (with a width of 250 μm) were patterned before the Au evaporation process step with an average laser power of 15% at a speed of 1,000 mm s^−1^ and frequency of 65 kHz for pulse duration of 120 ns. A 70 nm-thick Au layer was then deposited, and the P3 line (with a width of 40 μm) was made using the same scribing condition as the P2 line. The distance between P1 and P3 was around 370 μm, and a geometric fill factor (GFF) was around 93.7%.

### Characterization

The conductive atomic force microscope (C-AFM) was measured by Cypher ES Environmental AFM. UV–vis spectra are carried out on a UV–vis spectrophotometer (UV-3600 Plus, Shimadzu Co. Ltd, Japan). The current density–voltage (*J*-*V*) measurements were performed on a Keithley model 2400 digital source meter controlled by Test point software under a xenon lamp (450 W Xenon, AAA class). The light intensity was calibrated with a NREL-certified KG5-filtered Si reference diode. The active areas of small-size devices and modules were masked with a metal aperture of 0.06 and 29.0 cm^2^ (the dead area was included). X-ray diffraction (XRD) patterns were obtained by an X-ray diffractometer (XRD, Smartlab SE, Rigaku, Japan) with Cu K*α* radiation (*λ* = 0.15406 nm). PL mapping was carried out with a laser confocal Raman spectrometer (Princeton Instruments, Acton Standard Series SP-2558), a digital charge-coupled device (CCD) (PIXIS: 100B_eXcelon) and a 488-nm laser (PicoQuant LDH-P–C-485, 0.4 mW with a 1% optical density filter) using a homebuilt confocal microscope on a 20 μm × 20 μm sample area.

### Computational Methods

Density functional theory calculations used the Vienna Ab initio Simulation Package (VASP). The projected augmented wave (PAW) method and the Perdew–Burke–Ernzerhof (PBE) functional within the generalized gradient approximation (GGA) were employed to describe the interaction between ion-cores and valence electrons and exchange–correlation effects, and an energy cutoff of 500 eV was set for the plane-wave function expansion. The van der Waals (vdW) dispersion correction was described by the DFT-D3 correction. Transition states along the reaction pathway were determined using the nudged elastic band (NEB) method. The first Brillouin zone was sampled on a 3 × 3 × 1 gamma (Γ)-centered k-space mesh for structural relaxation and static calculations. The atomic positions of all slabs were relaxed until the total energy changes were less than 1.0 × 10^–5^ eV and the maximum force relaxed down to 0.05 eV Å^−1^. Calculations of transition states are based on the (001) perovskite surface.

The adsorption energy (*E*_ads_) is defined as *E*_ads_ = *E*_[4MBP]_^+^_/sur_–*E*_[4MBP]_^+^–*E*_sur_. *E*_[4MBP]_^+^_/sur_, *E*_[4MBP]_^+^ and *E*_sur_ are the energy of the [4MBP]^+^ cation adsorbed on the perovskite surface, [4MBP]^+^ and the perovskite surface, respectively.

## Results and Discussion

### Calculations and Model Analysis

**Fig. 1:**
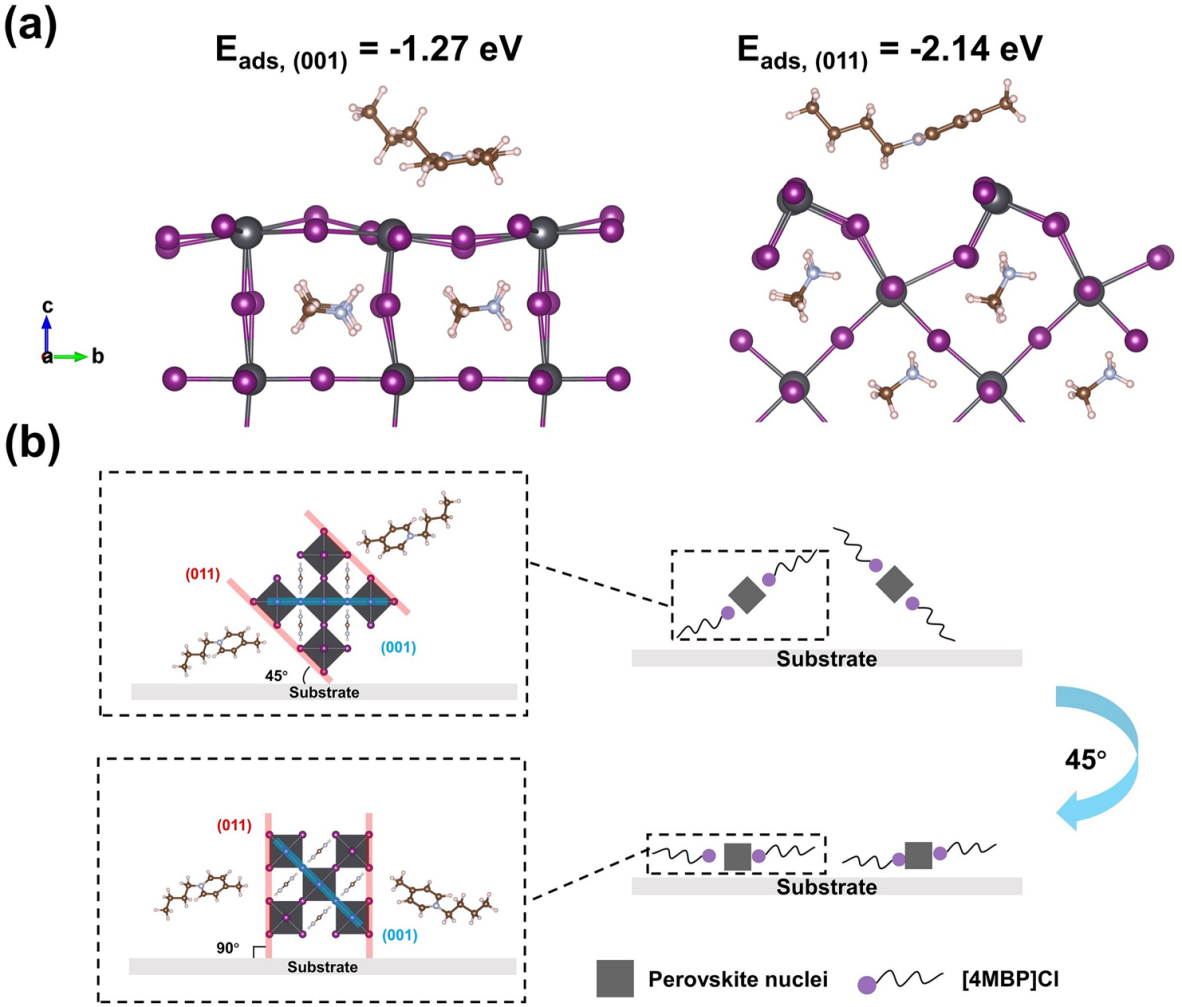
Calculations and model analysis. **a** Calculated models of [4MBP]Cl adsorbed on (001) and (011) facets of FAPbI_3_ and the corresponding adsorption energies. **b** Schematic of the proposed mechanism in which [4MBP]Cl changes the orientation of FAPbI_3_ nuclei to expose the (011) facet

In order to expose (011) facets, a dopant needs to selectively reduce the surface energy and growth rate of the (011) facet [20, 21]. As shown in Fig. 1a, the [4MBP]^+^ cation was placed on the (001) and (011) facets of perovskite to calculate the *E*_ads_, respectively. The calculated *E*_ads_ showed that the [4MBP]^+^ cation prefers to adsorb on the (011) facet because of |*E*_ads, (001)_| <|*E*_ads, (011)_| (1.27 vs 2.14 eV). The selective adsorption of [4MBP]^+^ lowers the surface energy of the (011) facet leading to slowing the growth of (011) plane due to the electrostatic interaction and steric hindrance provided by the [4MBP]^+^ cation [20, 23, 24]. The calculations indicate that the preferential adsorption of [4MBP]Cl will change the orientation of perovskite nuclei. As illustrated in Fig. 1b, the interaction of [4MBP]^+^ adsorbed on (011) facets should rotate perovskite nuclei by 45°. As a result, (011) crystal planes will stack along the out-of-plane direction, directly coming into contact with charge transport layers (Fig. S1), which is expected to accelerate charge carrier transport and collection [25, 26].

### Crystallization Kinetics and Orientational Preference Studies

**Fig. 2:**
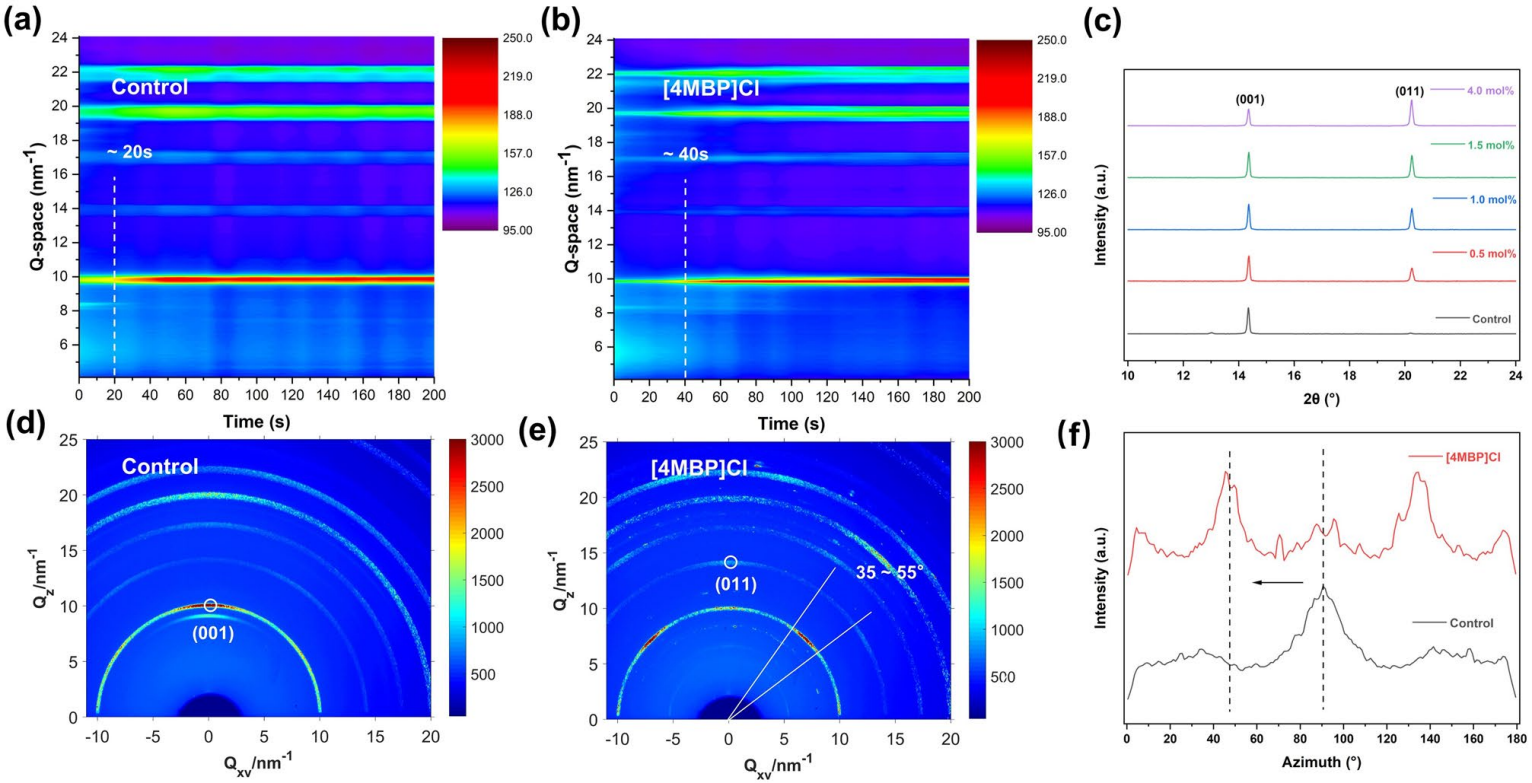
Crystallization kinetics and orientational preference studies of the FAPbI_3_ perovskite as a function of the [4MBP]Cl dopant. In-situ synchrotron radiation GIWAXS results of the FAPbI_3_ perovskite films **a** without and **b** with 4 mol% [4MBP]Cl. **c** XRD patterns of perovskite films containing different concentrations of [4MBP]Cl (0–4 mol%). 2D GIWAXS patterns of the FAPbI_3_ perovskite films **d** without and **e** with 4 mol% [4MBP]Cl. **f** Integrated GIWAXS intensity plots azimuthally along the ring at a *q* value approximate to 10 nm^−1^, assigned to the (001) plane of perovskite films without and with 4 mol% [4MBP]Cl

The role of chloride anions in enhancing carrier transport and stabilizing the perovskite structure has been extensively studied [17, 27]. To study the effect of the [4MBP]^+^ cation on the crystallization kinetics of FAPbI_3_ perovskite films, the films were doped with different concentrations of [4MBP]Cl (0–4 mol%). Perovskite films were analyzed using in situ grazing-incidence wide-angle X-ray scattering (GIWAXS). The GIWAXS intensity profiles of perovskites without [4MBP]Cl (control) and with [4MBP]Cl are plotted in Fig. 2a, b, respectively. For the [4MBP]Cl-doped film, the intensity of the (001) plane of FAPbI_3_ at a *q* value of 10 nm^−1^ (where *q* was the scattering vector, *q* = 4π sin *θ*/*λ*) increases after 40 s, which is 20 s later than the control film, indicating that [4MBP]Cl retards the growth rate of FAPbI_3_ perovskite. The slow growth of perovskite is beneficial in reducing defects and improving the quality of the film. Scanning electron microscopy (SEM) images reveal a significant increase in average grain sizes from ~ 0.6 to ~ 1.3 μm (Fig. S2). XRD measurements confirm that the (011) facet is exposed in the [4MBP]Cl-doped FAPbI_3_ perovskite film (Fig. 2c). As the amount of [4MBP]Cl dopant increases, the diffraction intensity of the (001) facet continuously decreases, whereas the diffraction intensity of the (011) facet increases. Notably, the positions of the diffraction peak remain unchanged, presumably because the [4MBP]^+^ cation is too large to enter into the lattice. Time-of-flight secondary ion mass spectrometry (ToF–SIMS) was carried out in positive ion mode to determine the cross-sectional distribution of the [4MBP]^+^ cation in PSCs. As illustrated in Fig. S3, the [4MBP]^+^ cation is mainly located at the interface between the compact SnO_2_ layer and the perovskite layer.

2D GIWAXS was used to probe the effect of the [4MBP]Cl dopant on the orientational preference of FAPbI_3_ perovskite films. For the control perovskite film, the (001) plane showed the out-of-plane direction due to the presence of MACl (Fig. 2d) [16–18]. In contrast, the orientational preference for (001) planes in the [4MBP]Cl-doped perovskite films changed from the out-of-plane to ~ 45°-oriented direction. Simultaneously, the (011) planes preferentially orientate in the out-of-plane direction (Fig. 2e). According to the integrated GIWAXS intensity plot azimuthally along the ring at a *q* value approximate to 10 nm^−1^ (Fig. 2f), the azimuth of the (001) plane decreases from 90° to ~ 45° in the [4MBP]Cl-doped perovskite film. XRD was used to probe the signal along the out-of-plane direction [28], which shows that the diffraction intensity of the (001) planes decrease as the [4MBP]Cl content increases (Fig. 2c).

### Influence of the (011) Facets on Carrier Dynamics

**Fig. 3:**
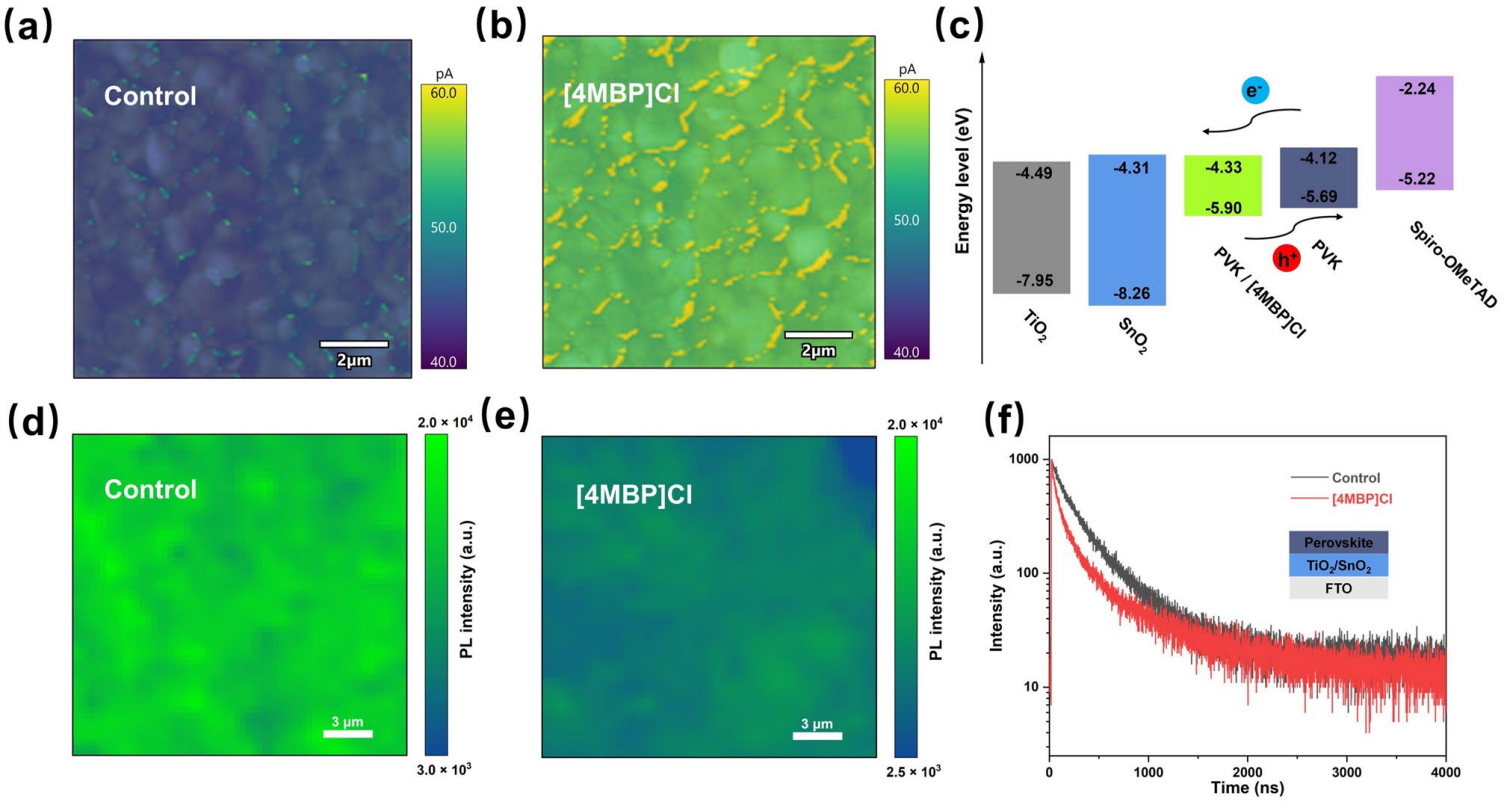
The influence of the (011) facets on carrier dynamics. C-AFM current mapping images of the FAPbI_3_ perovskite films **a** without and **b** with 0.5 mol% [4MBP]Cl. **c** Energy level diagram. PL mapping of the perovskite films **d** without and **e** with 0.5 mol% [4MBP]Cl deposited on FTO/ETL substrates. **f** TRPL spectra of the perovskite films without and with 0.5 mol% [4MBP]Cl deposited on FTO/ETL substrates

Small-size devices were fabricated using a planar architecture comprising FTO/c-TiO_2_/c-SnO_2_/perovskite/spiro-OMeTAD/Au. The device with the best performance was obtained with the film containing 0.5 mol% [4MBP]Cl. Increasing the concentration of [4MBP]Cl enhances the (011) orientation, but it can also slow the crystal growth process and enlarge the grain size, leading to the formation of nonuniform and uncompact perovskite films with many pinholes, as shown in Fig. S4. These features ultimately lead to a decrease in device performance. Therefore, it is important to optimize the (011) preferential orientation and perovskite film quality. The statistical results of small-size device’s parameters, short-circuit current density (*J*_sc_), open-circuit voltage (*V*_oc_), PCE and fill factor (FF) were shown in Fig. S5. The average *J*_sc_ of all the devices are similar (range 26.20–26.27 mA cm^−2^), which is consistent with the incident photon-to-current conversion efficiency (IPCE) results (Fig. S6). The devices with [4MBP]Cl-doped films have larger *V*_oc_ and FF values, which may be attributed to reduced defects and enhanced carrier transport. The champion device based on [4MBP]Cl-modified perovskite film achieved a *J*_sc_ of 26.29 mA cm^−2^, a *V*_oc_ of 1.151 V, a FF of 83.4% and a PCE of 25.24%. In contrast, the performance of the control device comprises a *J*_sc_ of 26.28 mA cm^−2^, a *V*_oc_ of 1.110 V, a FF of 83.1% and a PCE of 24.24% (Fig. S7). Further studies were therefore based on the perovskite film with the optimal [4MBP]Cl concentration of 0.5 mol% compared to the control device. The impact of the (011) facet on charge carrier dynamics was studied using conductive atomic force microscopy (C-AFM). Compared to the control film, the dark current is dramatically and uniformly enhanced in the [4MBP]Cl-modified perovskite film, with the average dark current increasing from 45.2 to 55.4 pA (Fig. 3a, b). This enhancement may be attributed to the good electrical properties of (011) facets as reported previously [14, 15]. Additionally, the current at the grain boundaries is higher than at the grains, presumably due to ionic conduction at the grain boundaries [29].

As expected, since the perovskite lattice remains unchanged, exposing the (011) facets does not influence the optical energy band gap (*E*_*g*_), confirmed by the UV–visible absorption spectra (Fig. S8). However, the (001) and (011) facets have different atomic arrangements, which causes a shift in the surface energy level of the perovskite. Ultraviolet photoelectron spectroscopy (UPS) was used to delineate the influence of the (011) facet on the surface energy level of perovskites (Fig. S9). The conduction band minimum (CBM) values decrease sharply from −4.12 to −4.33 eV following exposure of the (011) facets. As a result, the energy levels between the perovskite absorber and the electron transport layer (ETL) are better aligned, which facilitates electron extraction from the perovskite to the ETL (Fig. 3c). Energy level alignment is further confirmed by Kelvin probe force microscopy (KPFM). As shown in Fig. S10, the average contact potential difference (CPD) of grains in the control and [4MBP]Cl-modified perovskite film are 1.067 and 6.053 mV, respectively. Therefore, the (011) facet has a higher work function than the (001) facet in the perovskite films.

Steady-state photoluminescence (PL) mapping and time-resolved PL (TRPL) were used to further evaluate the effect of the (011) facets on charge carrier extraction and transport dynamics. The [4MBP]Cl-doped perovskite film deposited on FTO exhibited a higher PL intensity and a longer carrier lifetime than the control film, indicating the (011) facet inhibits non-radiative charge recombination (Fig. S11). The PL intensity of the [4MBP]Cl-modified perovskite film deposited on FTO/ETL is lower than that of the control perovskite film, demonstrating that the (011) facet promotes electron transfer from perovskite to ETL (Fig. 3d, e). As presented in Fig. 3f, compared with the control film, PL quenching of the [4MBP]Cl-doped film deposited on FTO/ETL is faster, indicative of a more efficient carrier extraction due to the better-matched energy level alignment.

A space-charge limited current (SCLC) model was used to characterize the density of trap states in the perovskite films. Dark current–voltage characteristics were collected on the electron-only devices (Fig. S12). The trap densities (*N*_*t*_) were extracted using the equation: ${N}_{t} =\frac{2\varepsilon {\varepsilon }_{0}{V}_{\mathrm{TFL}}}{e{L}^{2}}$, where *N*_*t*_ denotes the trap state density, *ε* and *ε*_0_ are the relative dielectric constant, and the vacuum dielectric constant respectively,* V*_TFL_ is the trap-filled limit voltage, *e* represents electron charge and *L* is the thickness of perovskite film [30]. *V*_TFL_ represents the bias voltage as the defects in the perovskite films were completely filled. Compared to the *V*_TFL_ value of the control film (0.203 V), the [4MBP]Cl-modified film has a smaller *V*_TFL_ value of 0.147 V. According to the equation, the trap densities decrease from 1.66 × 10^15^ to 1.43 × 10^15^ cm^−3^ in the [4MBP]Cl-doped film, attributed to the improved quality of the [4MBP]Cl-doped perovskite film. According to the equation, ${V}_{\mathrm{oc}} =\frac{nkT\mathrm{ln}I}{q}+$ *c* from the Shockley–Read–Hall recombination mechanism (where *T* is the absolute temperature, *k* is the Boltzmann constant, *q* is the elementary charge, *I* is incident light intensity, *c* is the constant and n represents an ideal factor), the closer the value of *n* to 1, the less trap-assisted non-radiational recombination exists in the device [31]. As presented in Fig. S13, the *n* value is 1.75 for the control device, decreasing to 1.39 in the [4MBP]Cl-modified device. Thus, non-radiative trap-dominated recombination is significantly suppressed due to the improved quality of perovskite films by [4MBP]Cl.

### Performance and Stability of the Photovoltaic Devices and Modules

**Fig. 4:**
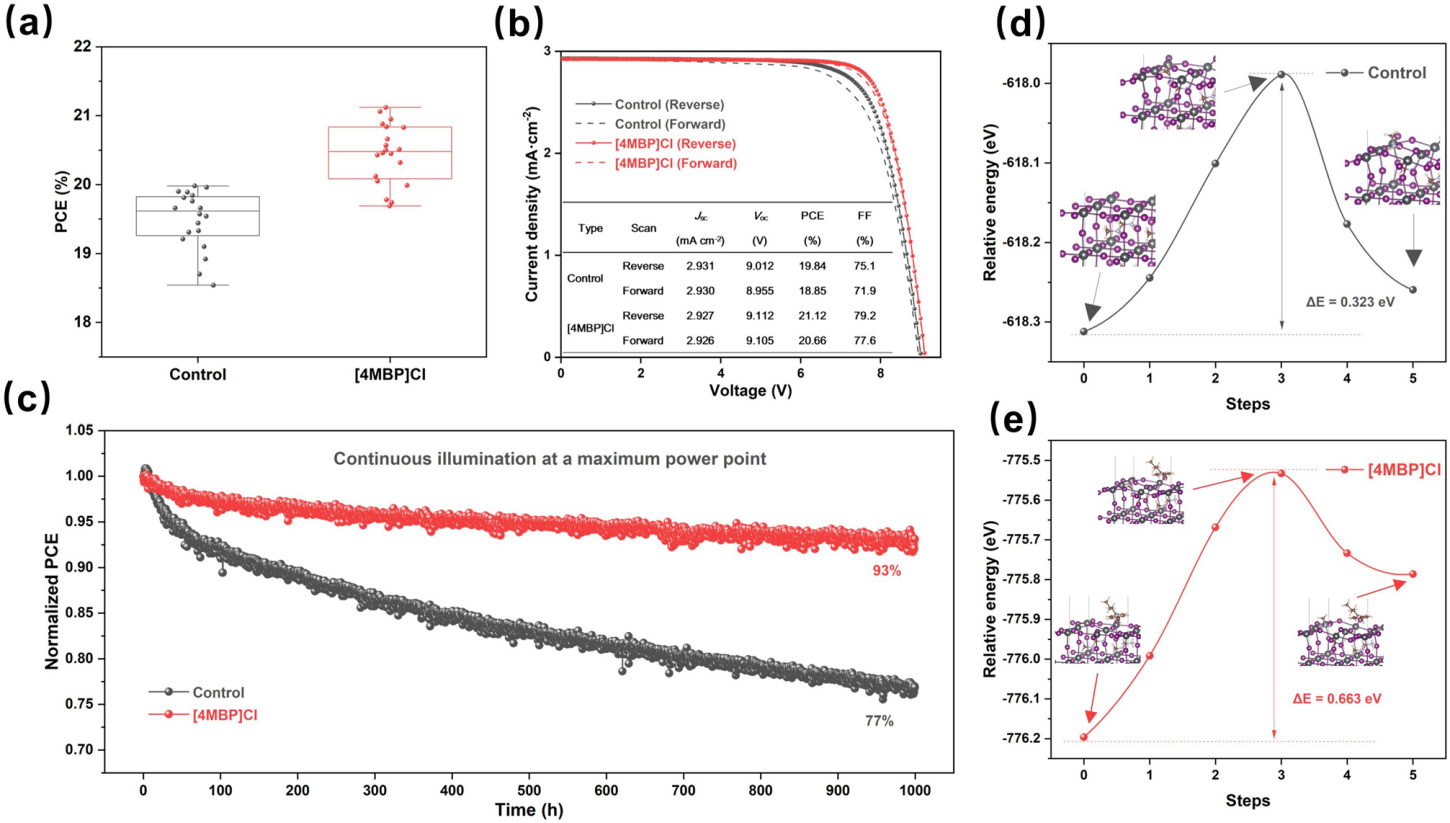
Performance and stability of the photovoltaic devices and modules. **a** Corresponding PCE distribution of modules fabricated without and with 0.5 mol% [4MBP]Cl. **b** *J*-*V* curves of modules fabricated without and with 0.5 mol% [4MBP]Cl. **c** Operation stability of devices fabricated without and with 0.5 mol% [4MBP]Cl under continuous illumination at a maximum power point. Energy profiles of MA^+^ ion migration in the perovskite **d** without and **e** with [4MBP]Cl. The structures of the NEB images at the initial, transition and final states are shown

Based on the better performance of the [4MBP]Cl-doped devices, 6.5 cm × 7.0 cm modules were fabricated using the vacuum quenching-assisted method [32]. The aperture area of modules is 29.0 cm^2^. Due to the improved carrier dynamics and perovskite film quality, the *V*_oc_ and FF of the [4MBP]Cl-doped modules are significantly enhanced compared to the control (Fig. S14). The average PCE increases from 19.50% for the control module to 20.45% for the [4MBP]Cl-doped module (Fig. 4a). The champion [4MBP]Cl-doped module has a PCE of 21.12% with a *J*_sc_ of 2.927 mA cm^−2^, a *V*_oc_ of 9.112 V and a FF of 79.2% in a reverse scan (RS), and a PCE of 20.66%, a *J*_sc_ of 2.926 mA cm^−2^, a *V*_oc_ of 9.105 V and a FF of 77.6% in a forward scan (FS), for comparison with the champion control module see Fig. 4b. The hysteretic index (HI = (PCE_Reverse_–PCE_Forward_)/PCE_Reverse_) decreases from 5.0% in the control module to 2.2% in the [4MBP]Cl-doped module, consistent with the reduced defects in the [4MBP]Cl-modified perovskite films.

The long-term operation stability was also studied by tracking the maximum power point under constant illumination (Fig. 4c). The [4MBP]Cl-doped device maintains 93% of its initial efficiency after 1,000 h, which compared favorably to the control device that retains only 77% of its initial efficiency. The increased photostability may be attributed to [4MBP]Cl located at grain boundaries, which could suppress ion migration and reduce defects.

The thermal stability of the PSCs was also probed with the unencapsulated devices and perovskite films heated at 60 °C under N_2_ and in the dark. The control devices rapidly deteriorate to 78% of the original PCE after 480 h. In contrast, the thermal stability of [4MBP]Cl-doped devices retains over 88% of the initial PCE after 864 h (Fig. S15). As shown in Fig. S15, the increased diffraction peak intensity of the control film at ca. 12.9° indicates that the perovskite decomposes to afford PbI_2_ when maintained at 60 °C for 480 h. However, decomposition is suppressed in the [4MBP]Cl-doped perovskite films. DFT calculations using the NEB method were performed to model the MA^+^ migration pathway and calculate the activation energy barrier (Δ*E*_*a*_) for ion migration (Fig. 4d–e) [33]. The Δ*E*_*a*_ of the control and [4MBP]Cl-doped perovskite was estimated to be 0.323 and 0.663 eV, respectively, indicating the inhibited decomposition by the [4MBP]Cl. The higher Δ*E*_*a*_ may be attributed to the electrostatic interactions and steric hindrance induced by [4MBP]Cl on the perovskite surface, which suppresses the migration of MA^+^ ions [24, 34].

## Conclusions

Exposure of the (011) facet of the MACl-modified FAPbI_3_ perovskite film using [4MBP]Cl has been shown to significantly enhance the charge transport ability of the perovskite film. The selective adsorption of the [4MBP]^+^ cation on the (011) surface was found to decrease the surface energy of (011) surface and slow the growth of (011) plane during perovskite crystallization process. This interaction of the [4MBP]^+^ cations also rotated the perovskite nuclei by 45°, leading to the (011) crystal planes stacking along the out-of-plane direction. Additionally, [4MBP]Cl greatly improved the stability of PSCs by reducing defects and increasing the activation energy barrier for the ion migration. The small-size device (0.06 cm^2^) and module (29.0 cm^2^) doped by the (011) facet achieved champion efficiencies of 25.24 and 21.12%, respectively. The [4MBP]Cl-doped device demonstrated good operational and thermal stability, indicating that this strategy could lead to more efficient and stable perovskite solar cells in future.

### Supplementary Information

Below is the link to the electronic supplementary material.

Supplementary file1 (PDF 2117 KB)
