# Supplementary material for: Highly Efficient and Stable FAPbI3 Perovskite Solar Cells and Modules Based on Exposure of the (011) Facet
Source: Nanomicro Lett. 2023 May 28;15:138. doi: 10.1007/s40820-023-01103-8 (PMC10225452; doi:10.1007/s40820-023-01103-8)
Supplement: Supplementary file 1 — Supplementary file1 (PDF 2117 KB) [file 40820_2023_1103_MOESM1_ESM.pdf]

Supporting Information for

Highly Efficient and Stable $FAPbI_3$ Perovskite Solar Cells and Modules Based on Exposure of the (011) Facet

Kai Zhang[1,2,†], Bin Ding[2,†], Chenyue Wang[3,†], Pengju Shi[4], Xianfu Zhang[1,2], Cheng Liu[1], Yi Yang[1], Xingyu Gao[3], Rui Wang[4,*], Li Tao[5,6,*], Keith G. Brooks[2], Songyuan Dai[1], Paul J. Dyson[2,*], Mohammad Khaja Nazeeruddin[2,*], and Yong Ding[1,2,*]

[1]Beijing Key Laboratory of Novel Thin-Film Solar Cells, North China Electric Power University, 102206 Beijing, P. R. China

[2]Institut des Sciences et Ingénierie Chimiques, Ecole Polytechnique Fédérale de Lausanne (EPFL), CH-1015 Lausanne, Switzerland

[3]Shanghai Synchrotron Radiation Facility (SSRF), Shanghai Advanced Research Institute, Chinese Academy of Sciences, 201204 Shanghai, P. R. China

[4]School of Engineering, Westlake University and Institute of Advanced Technology, Westlake Institute for Advanced Study, 310024 Hangzhou, P. R. China

[5]Hubei Yangtze Memory Laboratories, Wuhan 430205, People's Republic of China

[6]School of Microelectronics, Hubei University, Wuhan 430062, People's Republic of China

[†] Kai Zhang, Bin Ding, and Chenyue Wang contributed equally to this work.

*Corresponding authors. E-mail: wangrui@westlake.edu.cn (Rui Wang); litao@hubu.edu.cn (Li Tao); paul.dyson@epfl.ch (Paul J. Dyson); mdkhaja.nazeeruddin@epfl.ch (Mohammad Khaja Nazeeruddin); and dingy@ncepu.edu.cn (Yong Ding)

Supplementary Figures

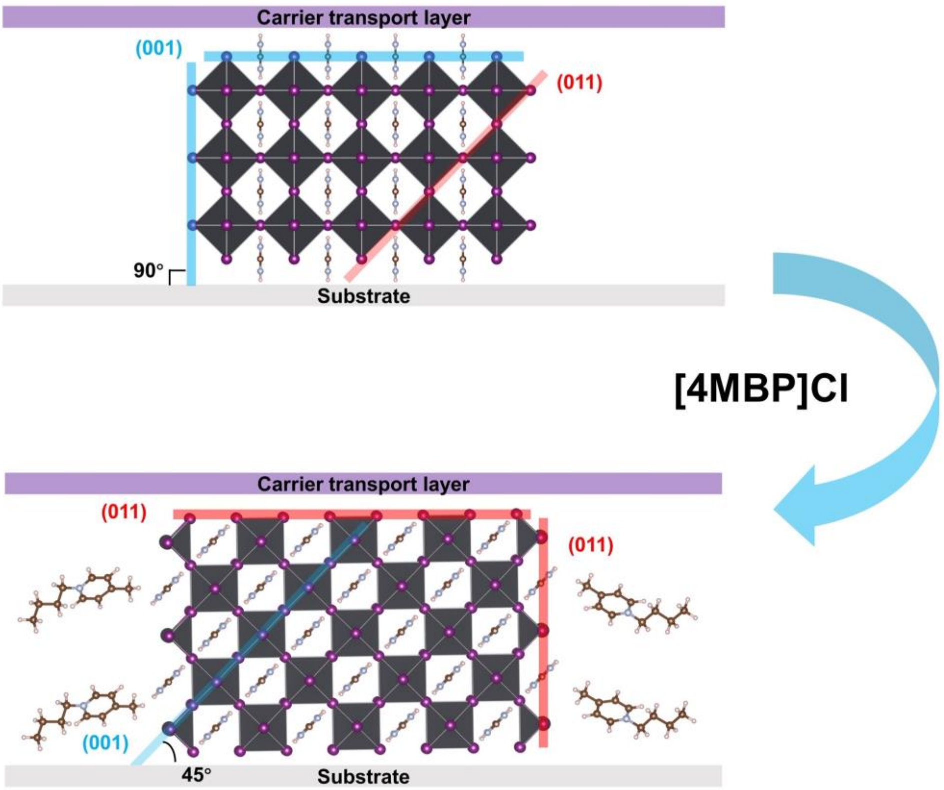

Fig. S1 Schematic showing the change in the stacking of (001) and (011) planes induced by [4MBP]Cl

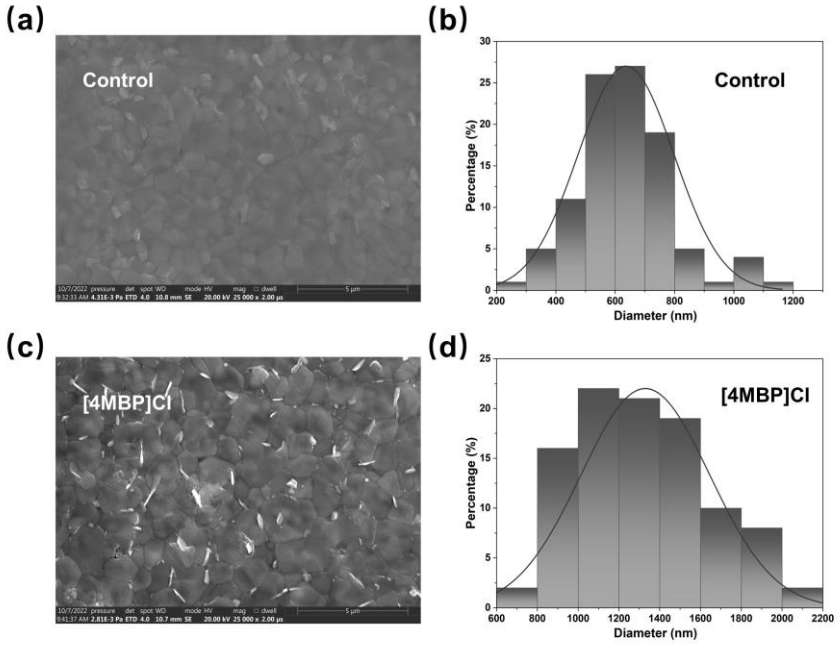

Fig. S2 Scanning electron microscopy (SEM) images and grain size distribution of perovskite films **a-b)** without and **c-d)** with 0.5 mol% [4MBP]Cl

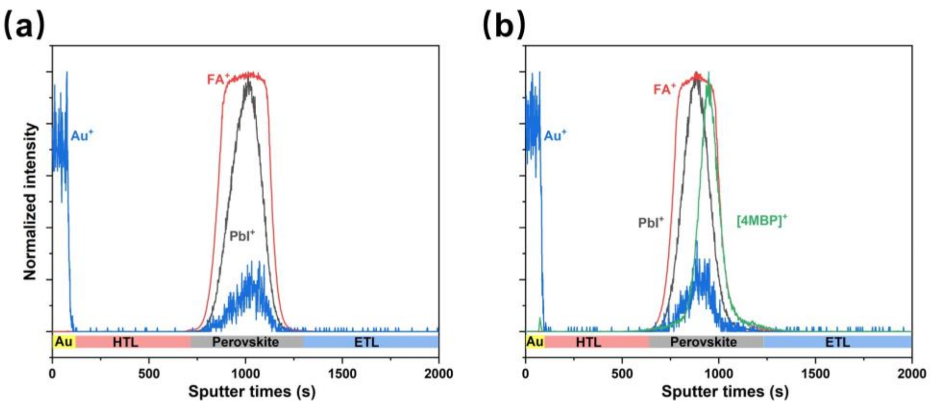

Fig. S3 Time-of-flight secondary ion mass spectrometry depth profiles for perovskite solar cells (PSCs) **a)** without and **b)** with 0.5 mol% [4MBP]Cl

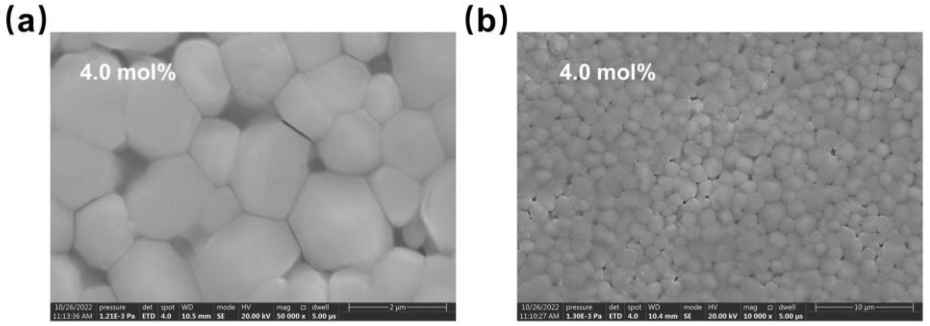

Fig. S4 SEM images of perovskite films with 4.0 mol% [4MBP]Cl under different magnifications, **a)** 50000X and **b)** 10000X

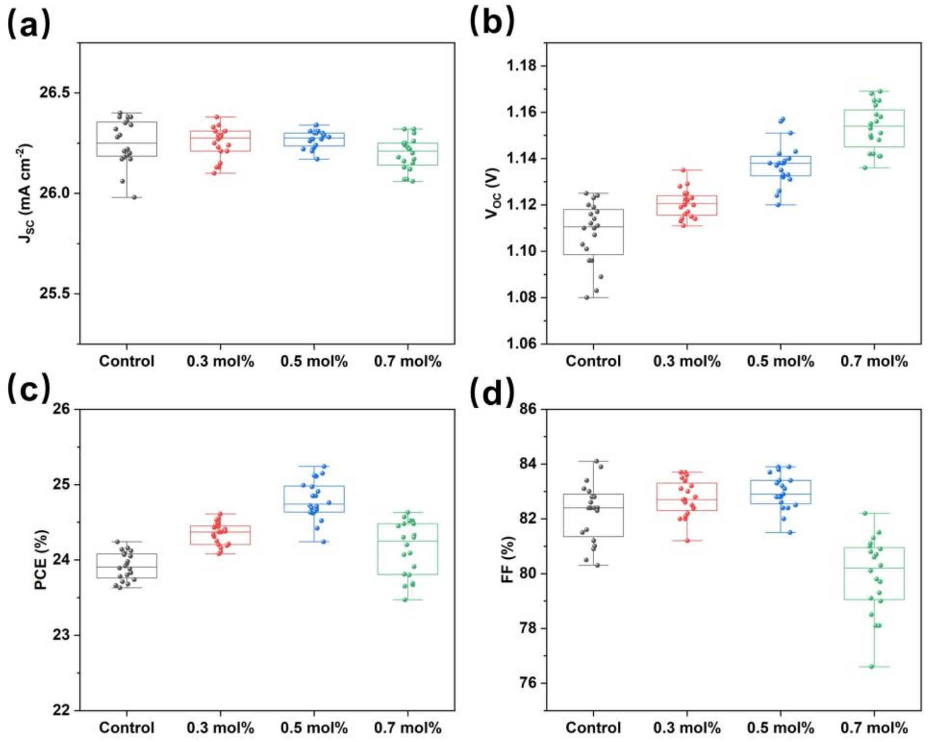

Fig. S5 a) J_{sc}, **b)** V_{oc}, **c)** PCE and **d)** FF of the control and [4MBP]Cl-doped devices

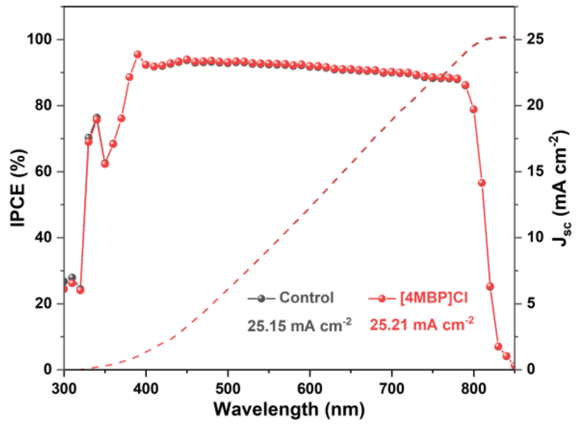

Fig. S6 IPCE of devices fabricated without and with 0.5 mol% [4MBP]Cl

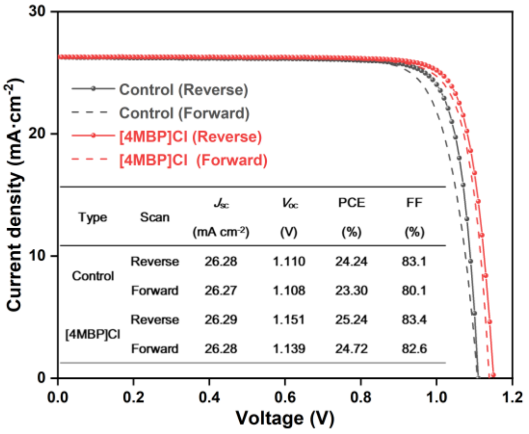

Type	Scan	J_{sc} (mA cm^{-2})	V_{oc} (V)	PCE (%)	FF (%)
Control	Reverse	26.28	1.110	24.24	83.1
	Forward	26.27	1.108	23.30	80.1
[4MBP]Cl	Reverse	26.29	1.151	25.24	83.4
	Forward	26.28	1.139	24.72	82.6

Fig. S7 *J-V* curves of devices fabricated without and with 0.5 mol% [4MBP]Cl

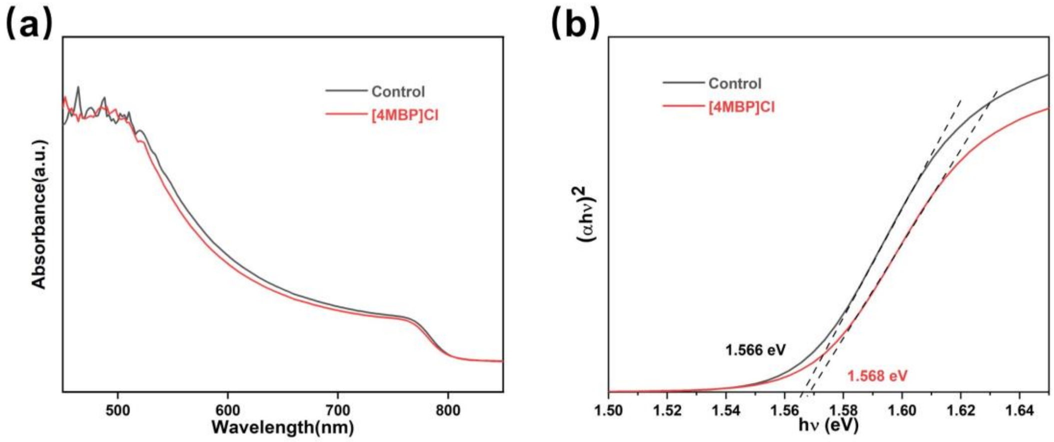

Fig. S8 a) UV−vis absorption spectra of perovskite films without and with 0.5 mol% [4MBP]Cl. **b)** Tauc plot of the corresponding UV-vis plot of the perovskite films without and with 0.5 mol% [4MBP]Cl

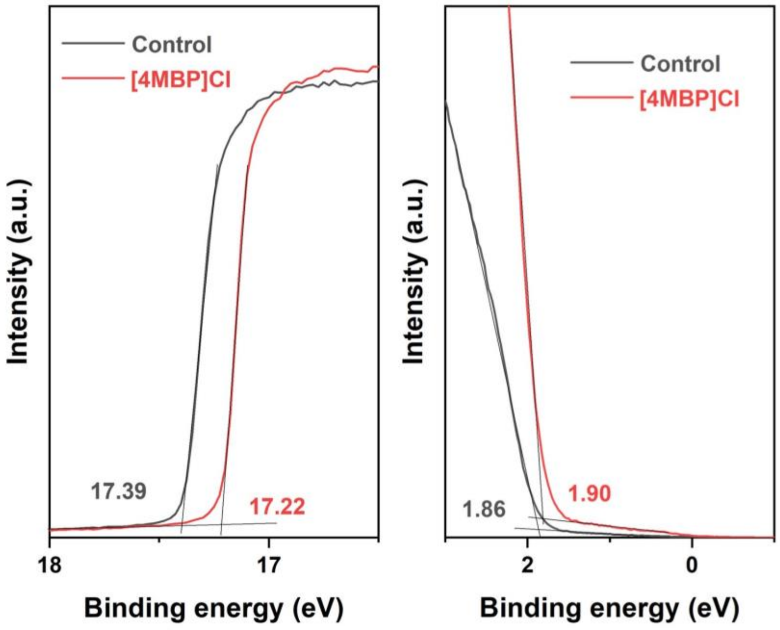

Fig. S9 UPS of perovskite films without and with 0.5 mol% [4MBP]Cl

The Fermi energy level (E_F) and valence band maximum (VBM) can be obtained using the following equations: $E_F = E_{cut\text{-}off}$ -21.22 (1) and VBM = $-E_F + E_{onset}$ (2). $E_{cut\text{-}off}$ and E_{onset} are 17.39 and 1.86 eV, respectively, for the control film. Similarly, $E_{cut\text{-}off}$ and E_{onset} of the [4MBP]Cl-modified film are 17.22 and 1.90 eV, respectively. The VBM of control and the [4MBP]Cl-doped films were calculated as -5.69 and -5.90 eV, respectively. Based on the optical energy band gap, the conduction band minimum (CBM) values for the control and [4MBP]Cl-modified film are -4.12 and -4.33 eV, respectively.

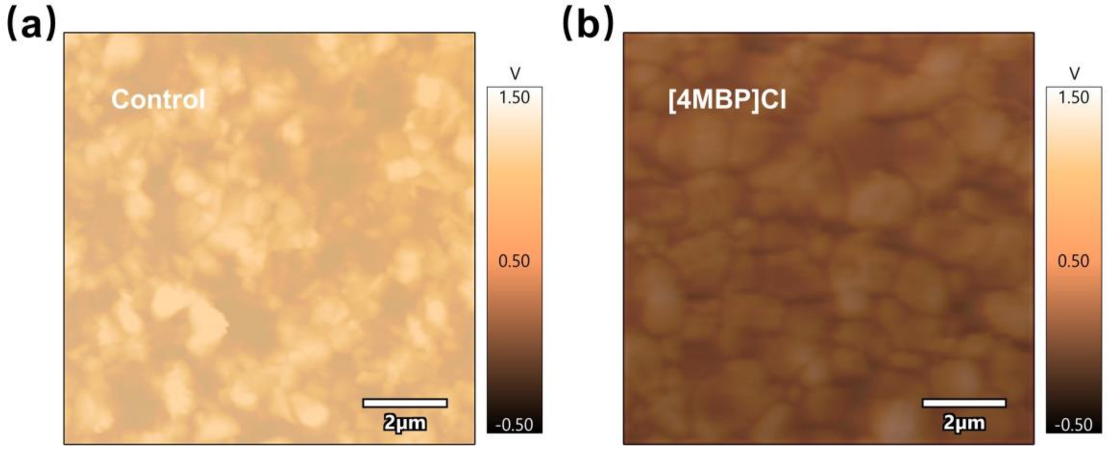

Fig. S10 KPFM images of perovskite films **a)** without and **b)** with 0.5 mol% [4MBP]Cl

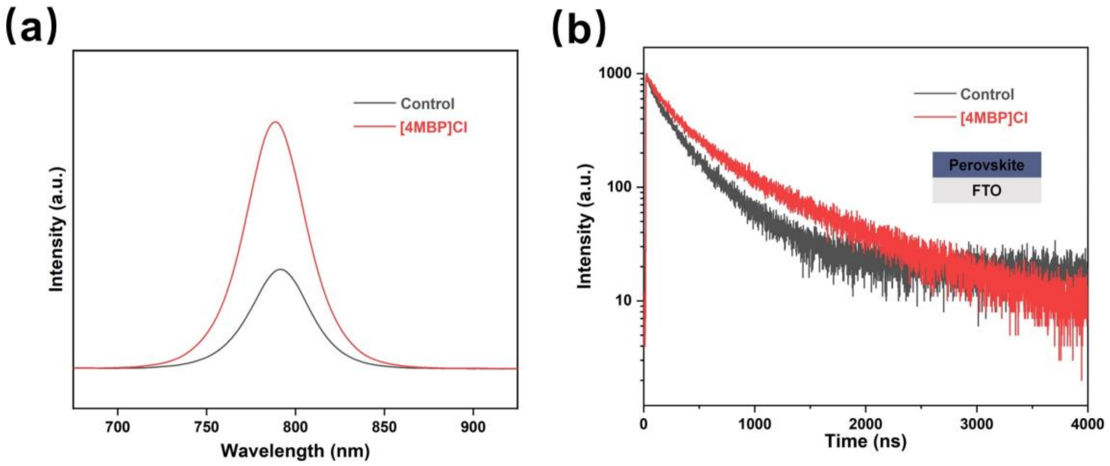

Fig. S11 a) PL spectra and **b)** TRPL spectra of perovskite films without and with 0.5 mol% [4MBP]Cl deposited on FTO substrates

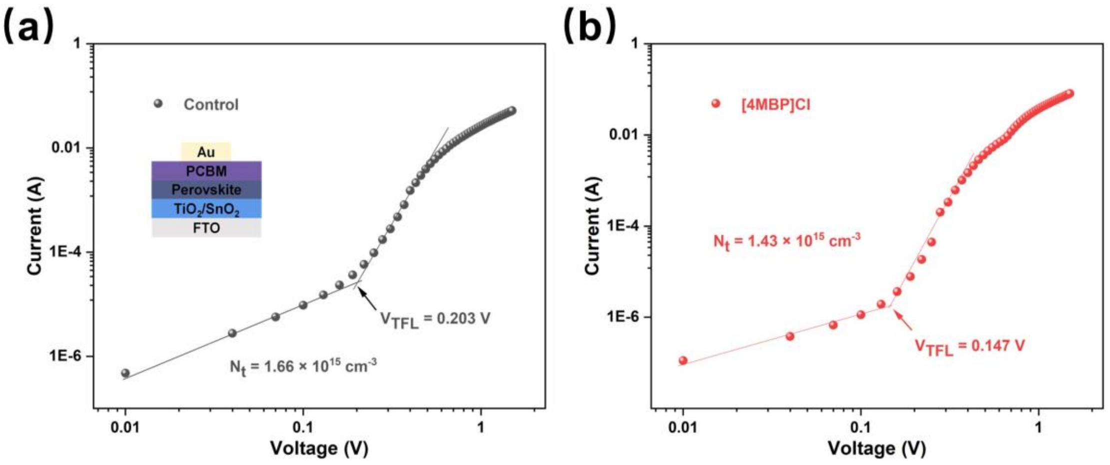

Fig. S12 SCLC curves of devices **a)** without and **b)** with 0.5 mol% [4MBP]Cl

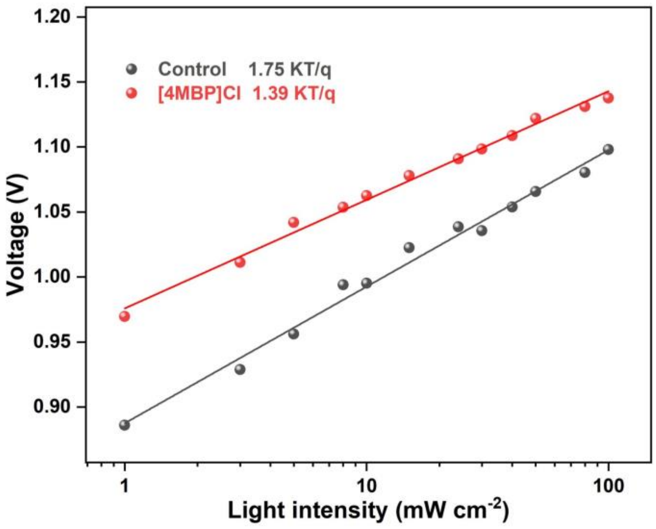

Fig. S13 V_{oc} dependence on the light intensity for the PSCs without and with 0.5 mol% [4MBP]Cl

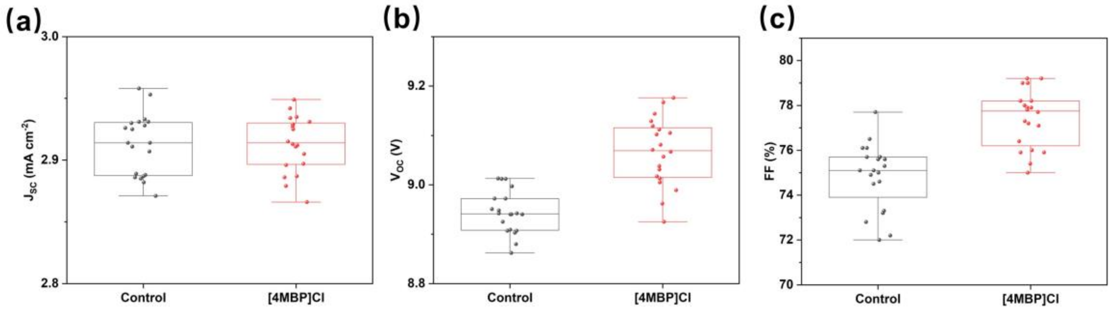

Fig. S14 a) J_{sc}, **b)** V_{oc} and **c)** FF of modules without and with 0.5 mol% [4MBP]Cl

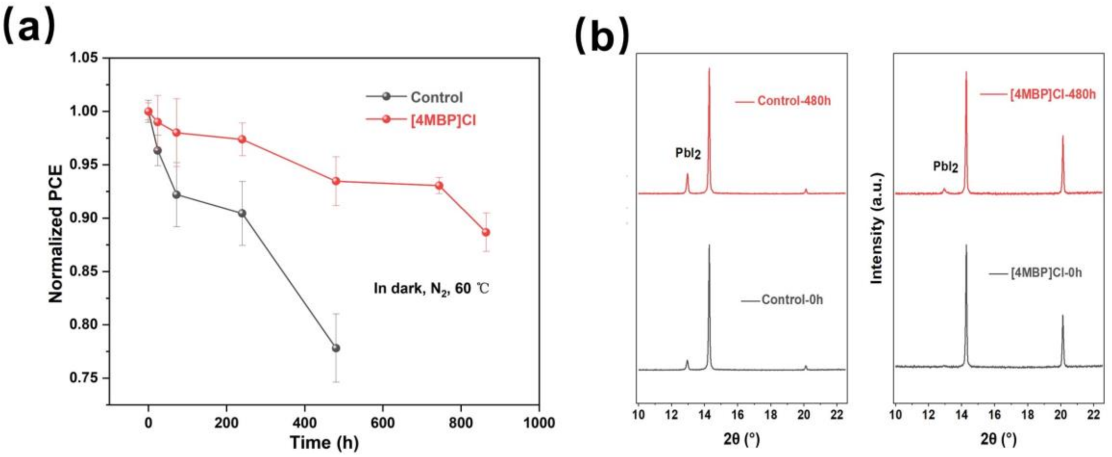

Fig. S15 a) PCE evolution of the unencapsulated PSCs without and with 0.5 mol% [4MBP]Cl at 60 °C under N_2 in the dark. **b)** XRD evolution of perovskite degradation without and with 0.5 mol% [4MBP]Cl at 60 °C under N_2 in the dark
